# Longitudinal diffusion and volumetric kinetics of head and neck cancer magnetic resonance on a 1.5 T MR-linear accelerator hybrid system: A prospective R-IDEAL stage 2a imaging biomarker characterization/pre-qualification study

**DOI:** 10.1016/j.ctro.2023.100666

**Published:** 2023-07-24

**Authors:** Joint Head and Neck Radiation Therapy-MRI Development Cooperative, Dina M. El-Habashy, Kareem A. Wahid, Renjie He, Brigid McDonald, Jillian Rigert, Samuel J. Mulder, Tze Yee Lim, Xin Wang, Jinzhong Yang, Yao Ding, Mohamed A. Naser, Sweet Ping Ng, Houda Bahig, Travis C. Salzillo, Kathryn E. Preston, Moamen Abobakr, Mohamed A. Shehata, Enas A. Elkhouly, Hagar A. Alagizy, Amira H. Hegazy, Mustefa Mohammadseid, Chris Terhaard, Marielle Philippens, David I. Rosenthal, Jihong Wang, Stephen Y. Lai, Alex Dresner, John C. Christodouleas, Abdallah Sherif Radwan Mohamed, Clifton D. Fuller, the MR-Linac Consortium Head and Neck Tumor Site Group

**Affiliations:** a Department of Radiation Oncology, The University of Texas MD Anderson Cancer Center, Houston, TX, USA; b Department of Clinical Oncology and Nuclear Medicine, Menoufia University, Shebin Elkom, Egypt; c Department of Radiation Physics, The University of Texas MD Anderson Cancer Center, Houston, TX, USA; d Department of Radiation Oncology, Austin Health Melbourne, Australia; e Department of Radiology, Radiation Oncology and Nuclear Medicine, Université de Montréal, Canada; f University of Houston College of Pharmacy, Houston, TX, USA; g Department of Radiation Therapy, University Medical Center Utrecht, Utrecht, The Netherlands; h Department of Head and Neck Surgery, Division of Surgery, The University of Texas MD Anderson Cancer Center, Houston, TX, USA; i Department of Molecular and Cellular Oncology, Division of Basic Science Research, The University of Texas MD Anderson Cancer Center, Houston, TX, USA; j Philips Healthcare MR Oncology, Cleveland, OH, USA; k Elekta AB, Stockholm, Sweden; l Department of Radiation Oncology, Baylor College of Medicine, Houston, TX, USA

**Keywords:** DWI, ADC, MR-Linac, Head and neck cancer, Oncologic outcomes, Diffusion

## Abstract

**Objectives:**

We aim to characterize the serial quantitative apparent diffusion coefficient (ADC) changes of the target disease volume using diffusion-weighted imaging (DWI) acquired weekly during radiation therapy (RT) on a 1.5 T MR-Linac and correlate these changes with tumor response and oncologic outcomes for head and neck squamous cell carcinoma (HNSCC) patients as part of a programmatic R-IDEAL biomarker characterization effort.

**Methods:**

Thirty patients with HNSCC who received curative-intent RT at MD Anderson Cancer Center, were included. Baseline and weekly MRI were obtained, and various ADC parameters were extracted from the regions of interest (ROIs). Baseline and weekly ADC parameters were correlated with response during and after RT, and the recurrence using the Mann-Whitney *U* test. The Wilcoxon signed-rank test was used to compare the weekly ADC versus baseline values. Weekly volumetric changes (Δvolume) for each ROI were correlated with ΔADC using Spearman’s Rho test. Recursive partitioning analysis (RPA) identified the optimal ΔADC threshold associated with different oncologic outcomes.

**Results:**

There was a significant rise in all ADC parameters at different time points of RT compared to baseline for both gross primary disease (GTV-P) and gross nodal disease volumes (GTV-N). The increased ADC values for GTV-P were statistically significant only for primary tumors achieving complete remission (CR) during RT. RPA identified GTV-P ΔADC 5th percentile > 13% at the mid-RT as the most significant parameter associated with primary tumors’ CR during RT (p < 0.001). There was a significant decrease in residual volume of both GTV-P & GTV-N throughout the course of RT. A significant negative correlation between mean ΔADC and Δvolume for GTV-P at the 3rd and 4th week of RT was detected (r = −0.39, p = 0.044 & r = −0.45, p = 0.019, respectively).

**Conclusion:**

Assessment of ADC kinetics at regular intervals throughout RT seems to be correlated with RT response. Further studies with larger cohorts and multi-institutional data are needed for validation of ΔADC as a model for prediction of response to RT.

## Highlights

• Serial DWI acquisition during RT showed that ADC parameters significantly increase throughout RT course especially for primary tumors achieving CR during treatment.
• For primary tumors developing CR during RT, the significant rise in mean ADC tended to appear starting from the 2nd week until the end of RT.
• Primary tumors’ ΔADC 5th percentile > 13% at mid-RT was associated with shorter time to CR.
• Dynamic ADC changes at a regular interval during radiation therapy (RT) can be a predictive imaging biomarker for early tumors’ response during RT.
• Future implications of these results may contribute to intra-treatment modification based on early response to RT, thus establishing the basis for MR-guided RT.

## Introduction

Head and neck squamous cell carcinoma (HNSCC) are the seventh most common malignancy worldwide [1], for which radiation therapy (RT) is considered the mainstay treatment [2]. Despite remarkable progress in RT techniques, treatment outcome is still unsatisfactory, especially for advanced stage HNSCC [3]. The ability to use a readily available, and easily interpretable biomarker to characterize tumors as resistant or sensitive, and adapt treatment accordingly remains an unmet need [4].

To date MRI has been shown potential to predict treatment response and oncologic outcomes [5]. While anatomical MRI has been used for accurate tumor definition, functional MRI sequences such as diffusion weighted imaging (DWI) can assess physiologic changes that may be undetected by the naked eye [6].

MRI linear accelerators (MR-Linacs) are innovative RT devices in which a linear accelerator is integrated with an on-board MRI scanner to enable MR-guided adaptive RT [7]. Therefore, acquisition of daily quantitative images which provide information regarding tissue characteristics and potential tumoral changes throughout the course of treatment, is now available [8].

Apparent diffusion coefficient (ADC), which is a quantitative parameter obtained from DWI, was studied as a potential imaging biomarker in head and neck cancer [9], [10], [11], [12], [13]. Our group has demonstrated that the primary tumors’ change in ADC at mid-RT is a robust predictor of oncologic outcomes in head and neck cancer [14]. Moreover, a recent study reported that DWI-guided dose painting had a better disease-free survival compared to conventional intensity-modulated RT (IMRT) in patients with locally advanced nasopharyngeal carcinoma [15]. Consequently, there is a great interest in using tumors’ ADC changes to adapt treatment plans based on early response, a process that can potentially be facilitated by high-frequency imaging on the MR-Linac. However, robust validation of DWI sequences on the MR-Linac is necessary, and to our knowledge, no studies in head and neck cancer have included DWI at a regular interval during RT using a MR-Linac device.

In this study, we aim to characterize the serial quantitative DWI changes in primary tumor and nodal target volumes in a pilot dataset of patients with HNSCC treated using the MR-Linac. According to the R-IDEAL framework, this study is considered a Stage 2a [16]. This work represents a unique opportunity to evaluate DWIs obtained throughout the entire RT course, to quantify ADC changes on a weekly basis, and to correlate these changes with response to RT and subsequent oncologic outcomes.

## Materials and methods

### Study population

Patients with HNSCC who received treatment with curative-intent IMRT using a 1.5 T MR-Linac (Elekta Unity; Stockholm, Sweden) at The University of Texas MD Anderson Cancer Center (MDACC), from May 16th, 2019 to February 22nd, 2021 were included in this study.

The study was approved by the Institutional Review Board (Protocol number: PA18-0341) at MDACC and patients provided an informed consent. The included patients are part of The Multi-OutcoMe EvaluatioN of radiation Therapy Using the Unity MR-Linac Study (MOMENTUM), which is a multi-institutional observational registry for patients treated on the MR-Linac system (NCT04075305).

Inclusion criteria were being at least 18 years old, having histologically confirmed HNSCC, having good performance status (Eastern Cooperative Oncology Group score of 0–2), receiving definitive RT for non-metastatic tumor, and having no contraindications for MRI [17].

### MR images

MRIs were acquired using the Unity system, which includes a Philips 1.5 T Marlin MRI with a 4-element anterior coil and a built-in 4 element posterior coil covering the head and neck region. MRIs were obtained for all patients at baseline and on a weekly basis throughout the RT course. The details of MRI sequences acquisition parameters are discussed in Appendix A, [18]

### Image segmentation and registration

**Fig. 1:**
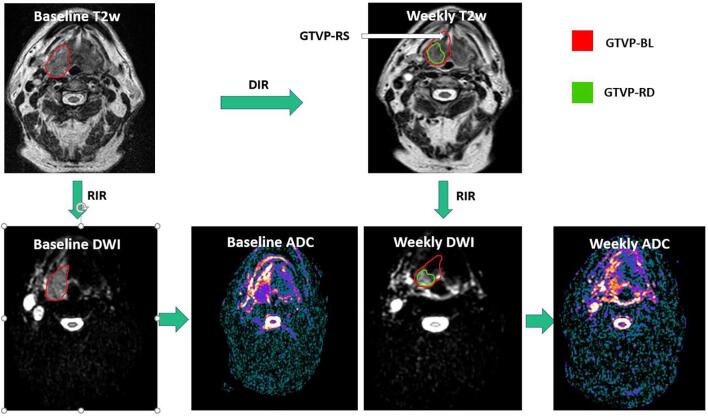
Image segmentation and registration workflow. T2w: T2 weighted DWI: Diffusion weighted image, ADC: Apparent diffusion coefficient RIR: Rigid image registration DIR: Deformable image registration GTVP-BL: Baseline primary tumor volume, GTVP-RD: The residual primary disease volume, GTVP-RS: The volume of the primary disease responding to RT.

Manual segmentation of the different region of interests (ROIs), including the gross primary disease volume (GTV-P) and gross nodal disease volumes (GTV-N) was performed by two expert radiation oncologists: DE and ASRM with 9 and 15 years of experience, respectively. The baseline primary disease volume, manually segmented on the baseline T2-weighted images, was labeled as GTVP-BL. The ROIs were propagated to the rigidly co-registered DWIs. Subsequently, deformable image registration was performed to co-register the images of different weeks with the baseline images. The baseline primary tumor volumes were then propagated from the baseline images to the co-registered weekly time points. For each weekly image, the residual primary disease volume was also manually segmented using the T2-weighted images and labeled as GTVP-RD. The response sub-volumes were created by subtracting the propagated initial GTVP-BL at each week minus the GTV-RD and was labeled as GTVP-RS. Propagation of all ROIs, from the T2-weighted images to the co-registered DWI of the same time point was done for all time points as illustrated in Fig. 1. The process of manual segmentation and registration was performed using the benchmarked commercially available image registration software Velocity AI (version 3.0.1). Finally, ADC measurements were extracted to assess ADC changes of the target volumes on a weekly basis relative to the baseline.

### Follow up and clinical outcomes

All the patients underwent physical examination, fiberoptic endoscopy, CECT, FDG PET-CT and MRI, performed 8 to 12 weeks after the end of RT for treatment response assessment. Response to treatment was defined as occurrence of complete remission (CR) or not based on the Response Evaluation Criteria in Solid Tumors, version 1.1. The evaluation of CR development during the course of RT was done using the MRIs acquired during treatment on MR-linac system. Time to CR, local control, recurrence free survival (RFS), and overall survival (OS) were the calculated endpoints.

### Statistical analysis

The collected data were tabulated and analyzed using SPSS version 26 and JMP pro, version 15. Continuous data were described as mean ± standard deviation (SD) and categorical data as proportions. The ADC values for all voxels of different ROIs were assessed by histogram analysis. The following ADC histogram parameters were extracted using an in-house MATLAB script (MATLAB, MathWorks, MA, USA): mean, 5th, 10th, 20th, 30th, 40th, 50th, 60th, 70th, 80th, 90th, and 95th percentiles. We examined the relationship between these ADC parameters (at the baseline and weekly during RT) versus treatment response (CR vs. non-CR)–during and after the RT course–and the development of recurrence, using the non-parametric Mann-Whitney *U* test. The non-parametric Wilcoxon signed-rank test was used to compare the weekly ADC histogram parameters to baseline values. We used Bonferroni correction for multiple comparison across different weeks and p < 0.008 was considered significant. The ΔADC, which represents the percent change of the ADC histogram parameter at each week (ADC_wk) of RT course relative to the baseline value for each ADC parameter (ADC_BL), was calculated using the following equation: ($\frac{ADC\_wk-ADC\_BL}{ADC\_BL}\times 100\%$). Volumetric changes for both GTV-P and GTV-N at weekly RT were calculated, and the non-parametric Spearman rho test was used to determine the relationship between ΔADC and change in volume. Recursive partitioning analysis (RPA) was performed to identify ΔADC threshold associated with different oncological outcomes.

Missing ADC parameter values (due to a few patients with missing weekly images) were imputed based on the remaining timepoints available for a given patient using an order-1 linear spline method performed using an in-house Python script (Python version 3.8.8).

In this work, we followed the checklist of items to be reported per the REporting recommendations for tumor MARKer (REMARK) guidelines for prognostic studies [19].

## Results

### Patient and disease characteristics

Thirty patients with HNSCC were included in this study; most patients were male (n = 28, 93.3%). At baseline, 18 patients had both primary tumor and lymph node involvement. Nine patients exhibited a primary tumor without lymph node involvement, and 3 patients had nodal disease only. Those who did not have primary tumor had either carcinoma of unknown primary (CUP) or had their primary tumor surgically resected (i.e., tonsillectomy). Oropharyngeal carcinoma represented the most common type of HNSCC (n = 21, 70%), followed by laryngeal cancer (n = 7, 23.3%). Twenty-two patients (73.3%) had positive test results for HPV, with 20 patients having oropharyngeal primary tumors, 1 patient having CUP, and 1 patient having laryngeal carcinoma. Twenty-five patients (83.3%) had early-stage tumors (stage I and II), as classified by the American Joint Committee on Cancer guidelines, 8th edition. A summary of the patients’ and disease characteristics are shown in Table 1.

**Table 1:** Patients’ and Disease Characteristics:

Patient characteristics	Mean ± SD, range
**Age (years)**	64.1 ± 10.37, 37–82
**Gender**	
Male	28 (93.3%)
Female	2 (6.7%)
**Anatomical location**	
Oropharynx	21 (70%)
Larynx	7 (23.3%)
Hypopharynx	1 (3.3%)
CUP	1 (3.3%)
**TNM Stage**	
I	21 (70%)
II	4 (13.3%)
III	3 (10%)
IV	2 (6.7%)
**T stage**	
Tx	1 (3.3%)
T1	13 (43.3%)
T2	13 (43.3%)
T3	1 (3.3%)
T4	2 (6.7%)
**N stage**	
N0	9 (30%)
N1	14 (46.7%)
N2	6 (20%)
N3	1 (3.3%)
**HPV status**	
Positive	22 (73.3%)
Negative	1 (3.3%)
unknown	7 (23.3%)
**Smoking Status**	
Smoker	1 (3.3%)
Non-smoker	15 (50%)
Ex-smoker	14 (46.7%)
**Surgery for the primary**	
Yes	7 (23.3%)
No	23 (76.7%)
**RT dose (Gy)**	68.5 ± 2.45, 63–70
**Number of fractions**	32.2 ± 1.93, 28–35
**Chemotherapy**	19 (63.3%)
Induction CT	1 (3.3%)
Concurrent CT	18 (60%)
**CR for the primary during RT course**	
Yes	11 (36.7%)
No	19 (63.3%)
**CR at the end of RT**	
Yes	26 (86.7%)
No	4 (13.3%)
**Recurrence**	
Yes	5 (16.7%)
No	25 (83.3%)
**Type of recurrence**	
Local	1
Regional	0
Distant	4

### Oncologic outcomes

The median follow-up time for the patients was 19.6 months (range, 11.5–39.8 months), with 2-year local control, RFS and OS rates of 86. 7%, 83.3%, and 96.7%, respectively. Throughout the RT course, 11 of the 27 patients who had GTV-P had a CR for the primary tumor. Of these 11 patients, 5 patients had a CR at the 6th week of RT, 3 at the 5th week, 2 at the 4th week, and 1 at the 3rd week. Twenty-six patients (86.7%) had a CR of both the primary tumor and the metastatic lymph nodes when evaluated 8 to 12 weeks after the end of RT. No patients had a CR for the lymph node metastases during RT. During surveillance, recurrence occurred for 5 patients (16.6%), with only one had local recurrence and 4 patients had distant recurrence. Among the patients who experienced distant recurrence, two of them achieved CR for the primary tumor during RT. However, these two patients failed to achieve radiological CR for the metastatic lymph nodes after the completion of RT. In one case, both the primary tumor and metastatic lymph nodes achieved CR only after the end of RT. Conversely, in another case, neither the primary tumor nor the metastatic node achieved CR either during or after the completion of RT. For the patient who developed local recurrence, radiological CR was attained only after the conclusion of RT. All patients had their recurrence pathologically confirmed.

### Analysis of ADC kinetics

All studied pre-treatment ADC parameters did not have a significant association with the response to RT or the oncologic outcomes (p > 0.05).

**Fig. 2:**
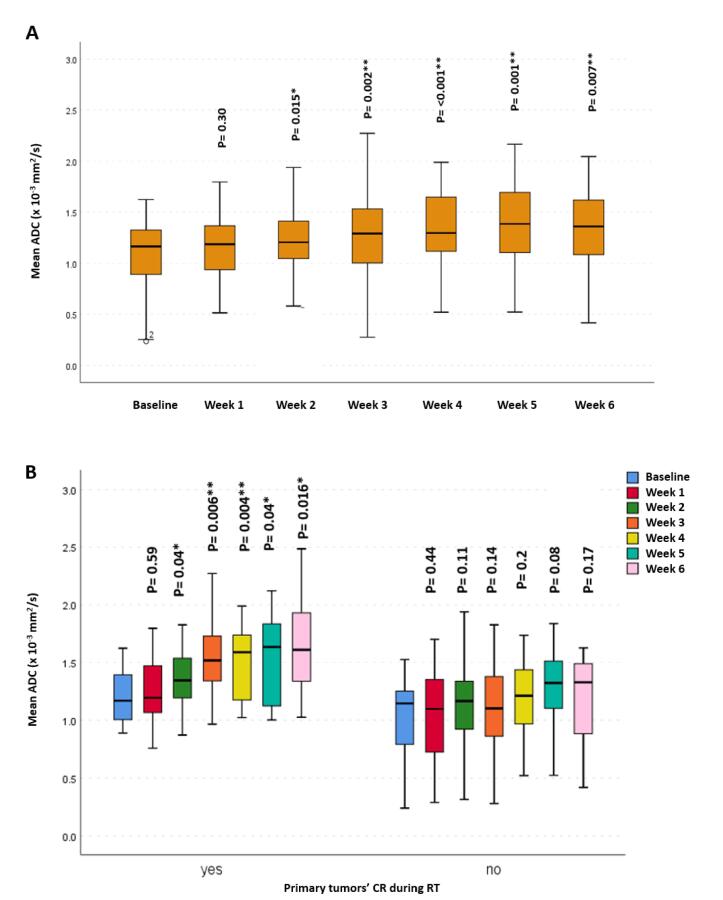
Mean apparent diffusion coefficient (ADC) at different timepoints for GTV-P: (A) For all patients, (B) patients who developed CR during RT versus those who did not.

**Fig. 3:**
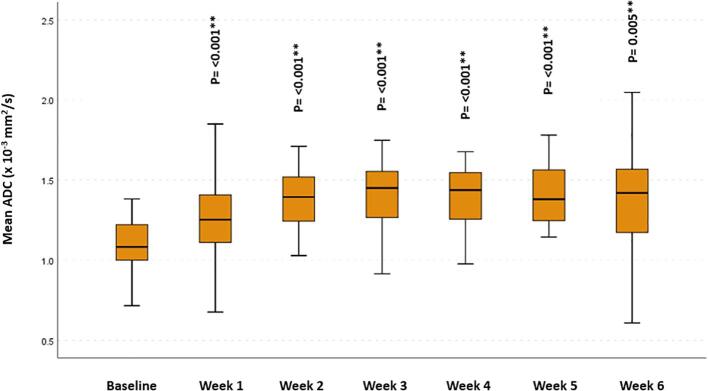
Mean ADC at different timepoints for GTV-N. * Significance before Bonferroni correction, ** Significance after Bonferroni correction.

Overall, there was a significant increase, starting from the 2nd week of RT, in the propagated initial GTV-P mean ADC, compared to baseline mean ADC (p = 0.015, 0.002, <0.001, 0.001 and 0.007, respectively). However, this increase in mean ADC reached statistical significance only for primary tumors developing CR during RT (p = 0.04, 0.006, 0.004, 0.04 and 0.016, respectively). Similarly, a statistically significant increase in GTV-N mean ADC compared to the baseline mean ADC (p < 0.001 and 0.005, respectively) was found in all patients (Fig. 2, Fig. 3).

**Fig. 4:**
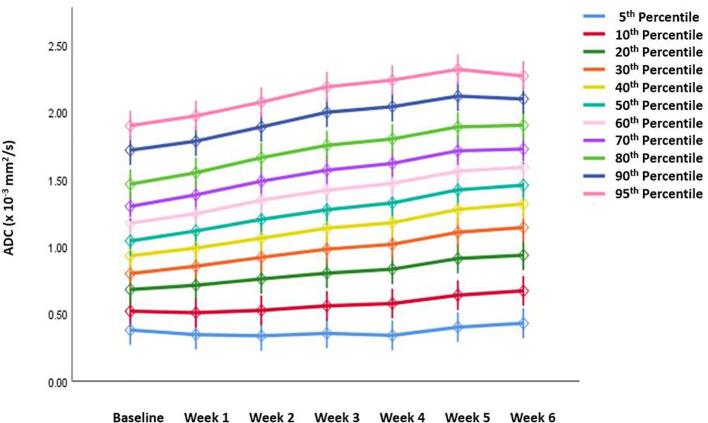
Absolute ADC histogram parameters for GTV-P across different time points.

All other ADC parameters for the GTV-P showed an incremental increase from the baseline throughout the course of RT (Fig. 4).

**Fig. 5:**
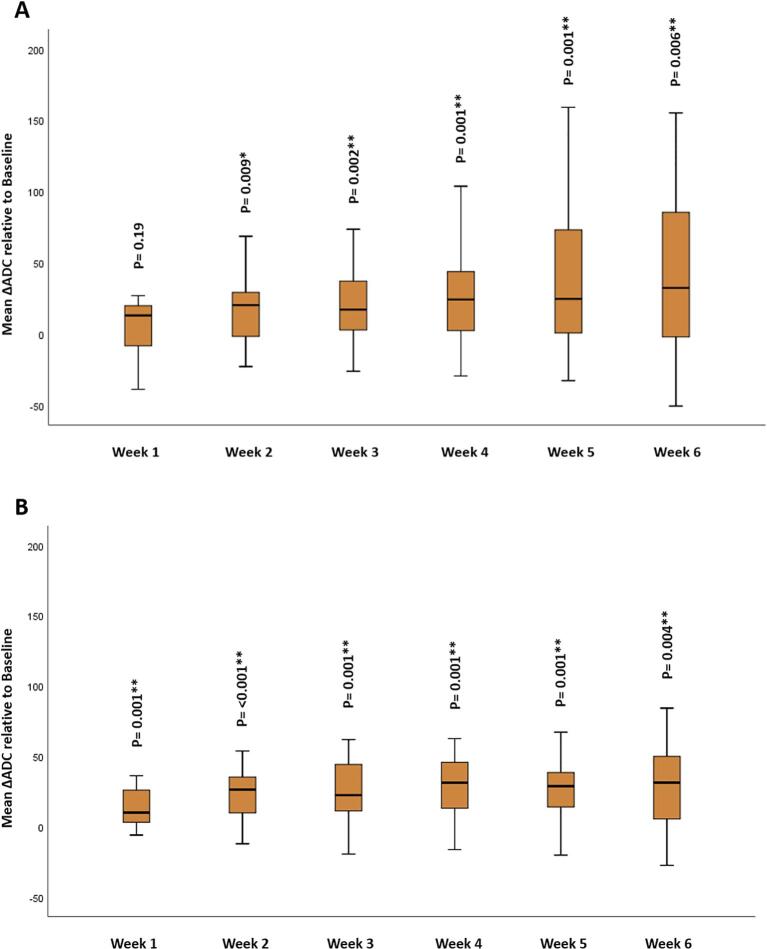
Mean ΔADC change across different timepoints for: (A) GTV-P, (B) GTV_N * Significance before Bonferroni correction, ** Significance after Bonferroni correction.

There was a significant rise for the ΔADC and the initial mean values for both the primary tumor and metastatic lymph nodes during the course of RT. The significant increase in ΔADC for the primary tumor was observed starting from the second week of RT (with corresponding p-values of 0.19, 0.009, 0.002, 0.001, 0.001, and 0.006, respectively). In contrast, the significant increase in ΔADC for the metastatic lymph nodes was observed as early as the first week of RT (p = 0.001, < 0.001, 0.001, and 0.004, respectively; Fig. 5).

A significant association was detected between ΔADC 5th, 10th, 20th, 30th, 40th, 60th and 70th percentiles at the 3rd week of RT for the propagated baseline GTV-P, and occurrence of CR for the primary tumor, during RT (n = 11, 40.7%) (p = 0.029, 0.036, 0.042, 0.049, 0.048, 0.034, and 0.048, respectively). RPA identified a ΔADC 5th percentile > 13% at the 3rd week of RT as the most significant parameter associated with shorter time to CR for the primary tumor during RT (p < 0.001). However, there was no statistically significant correlation between GTV-P ΔADC parameters at various time points and either CR or recurrence after the end of RT.

No significant correlation was found between mean ΔADC for GTV-N and other oncologic outcomes.

### Analysis of volumetric and mean ΔADC changes

**Fig. 6:**
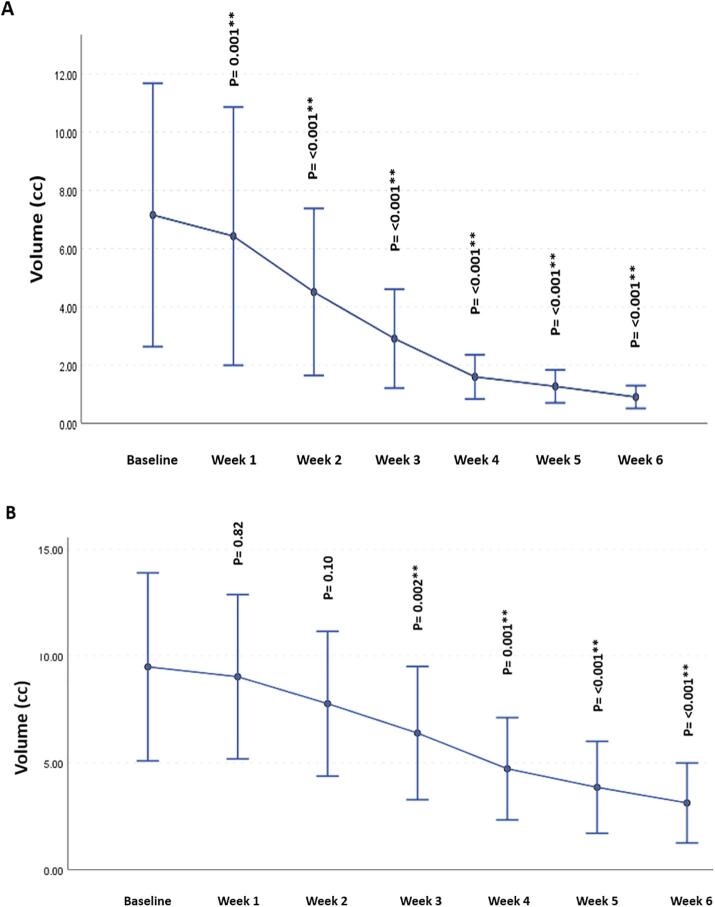
Volumetric changes in GTVP-RD (A) & GTV-N (B) throughout the course of radiation therapy, ** Significance after Bonferroni correction.

There was a significant decrease in residual tumor volumes for both GTV-P and GTV-N compared to baseline volumes at different time points throughout the RT course. Although this gradual decrease in the residual primary tumors’ volume was detected earlier, starting from the 1st week of RT (p = 0.001 and < 0.001, respectively), the GTV-N volumetric decline was first demonstrated at week 3 (p = 0.002, 0.001 and < 0.001, respectively; Fig. 6).

**Fig. 7:**
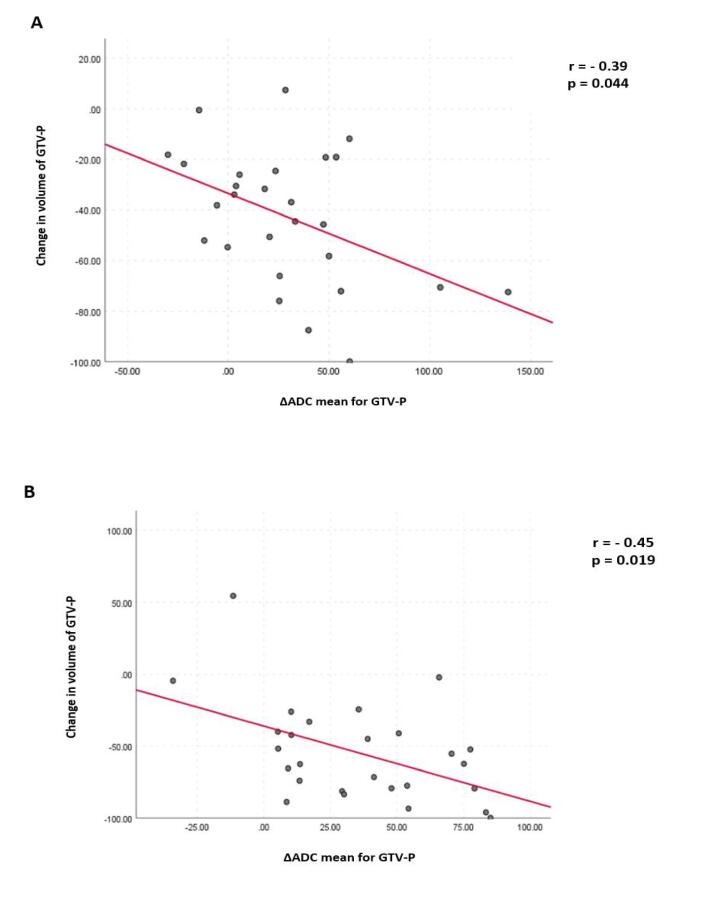
The relationship between changes in volume and mean ADC for GTVP-RD: (A) at the 3rd week (B) at the 4th week of radiation therapy.

A statistically significant negative correlation was demonstrated between the mean ΔADC and change in volume for GTVP-RD at the 3rd and 4th weeks of RT (r = − 0.39, p = 0.044, and r = − 0.45, p = 0.019, respectively; Fig. 7). However, no significant correlation was found between mean ΔADC and volumetric changes for GTV-N (p > 0.05).

A significant association was detected between change in volume for GTVP-RD at the 4th, 5th, and 6th week of RT and occurrence of CR for the primary tumor during RT (p = 0.003, 0.001 and 0.003, respectively). However, there was no significant correlation between volumetric changes, either in GTV-P or GTV-N, and the different oncologic outcomes at the end of RT (p > 0.05).

### ROI sub-volume analysis

As detected for the propagated initial GTV-P, there was an incremental increase in GTVP-RD mean ADC compared to the baseline values. This rise in GTVP-RD mean ADC was first demonstrated at the 2nd week of RT.

A significant correlation was detected between mean ΔADC at the 3rd week of RT for GTVP-RD and the primary tumors’ CR, during RT (p = 0.043). RPA identified a mean ΔADC > 20% at the 3rd week of RT as the most significant parameter associated with shorter time to CR (p = 0.013).

### HPV status subgroup analysis

Appendix B

## Discussion

In this prospective study, quantitative DWIs obtained weekly throughout the RT course were used to determine the gross tumors’ ADC kinetics, in a granular form during RT. These ADC changes were correlated with response during and after RT and oncologic outcomes.

Our study demonstrated a statistically significant rise in ADC parameters during RT compared to baseline values in all patients. The increased mean ADC values, starting from the 2nd week during RT, were found to be higher in patients who developed radiologic CR for the primary tumor during RT. These findings match the findings of previous studies highlighting the correlation between ADC changes and response to RT in HNSCC [10], [20]. The exact timing of these ADC changes was not clear in the previous publications. One of the most important findings in our study is that GTV-P mean ADC for patients with a favorable response to RT started to significantly increase at the 2nd week of treatment and continued until the end of RT. In the same context, we observed a significant increase in the mean ADC for the metastatic lymph nodes at various timepoints during RT when compared to the baseline mean ADC. It is noteworthy that this increase reached its peak at the 6th week of RT. This finding can potentially be explained by the development of necrosis and cystic degeneration within the metastatic lymph nodes as RT progresses. These factors contribute to an increased cystic component within the metastatic lymph nodes, resulting in higher ADC values as RT progresses.

Additionally, in our pilot data, the most significant correlation between the primary tumors’ ΔADC at different parameters and CR of the primary tumor during RT appears to be noticeable at the 3rd week of treatment. RPA has identified a 13% increase in GTV-P ΔADC 5th percentile at the 3rd week of RT relative to the baseline as the most significant factor for predicting CR for the primary tumor during RT. This is in agreement with a recent study conducted by our group showed an increase of 7% in the GTV-P mean ΔADC at mid-RT in comparison to the baseline was significantly associated with better locoregional control [14]. The identification of tumors developing CR during RT may be associated with better local control and oncologic outcomes in head and neck cancer patients as suggested in previous studies [21], [22]. Although a significant correlation between higher ΔADC parameters and the occurrence of CR for the primary tumor during RT was detected, no significant correlation was found between ΔADC and either the response post-treatment or recurrence. This is possibly due to the small number of patients who did not achieve CR after the end of RT or experienced recurrence at a later stage. These findings contradict the previous data showing a significant association between ΔADC and both locoregional control and RFS at the end of RT [23], [24]. The previously described ADC changes were assessed for the GTV-P propagated unchanged from the baseline to weekly MRIs after image registration. This means that these ΔADC parameters were independent from the volumetric changes in the primary tumor. In terms of primary residual tumor (GTVP-RD), RPA found a ΔADC mean more than 20%, at the 3rd week of RT to be significantly correlated with a shorter time to CR for the primary tumor during RT.

Our data could not reach a conclusion regarding the association of serial ADC changes and endpoints because of the nature of this relatively small pilot study with a limited number of events. There was no significant association between the ADC parameters, extracted from GTV-N and loco-regional control or RFS. This finding might be explained by the absence of regional recurrence in our cohort. Moreover, the heterogeneity of the lymph node metastases (presence of cystic and solid components) may contribute to the absence of correlation between the nodal ADC parameters and oncologic outcomes.

We also showed that baseline ADC parameters, whether in the primary tumor or lymph node metastases, had no significant correlation with different oncologic outcomes, indicating that dynamic information obtained from RT-induced imaging changes during treatment is likely more informative compared to baseline values. This finding is in agreement with previous studies by our group as well as by other groups [14], [25], [26]. However, these findings conflict with some studies that showed a significant association between pre-treatment ADC parameters and the response to treatment [27], [28].

The volumetric analysis for different ROIs, showed a significant decrease in the GTVP-RD volume during RT compared to the baseline volume. This volumetric decrease was observed immediately starting from the 1st week of RT. However, a delayed decremental decline in GTV-N volumetric changes, starting from the 3rd week of RT, was demonstrated. This finding may justify the failure of lymph node metastases to attain CR during RT in contrast to the higher rate of intra-treatment CR achieved by primary tumors.

Another important finding in our study, is the significant negative correlation that was found between mean ΔADC and volumetric changes of GTVP-RD at the 3rd and 4th weeks of RT however, no significant correlation was detected between changes in both mean ADC and volume of GTV-N. This finding agrees with similar findings by Ng at al [29].

Similar profile of imaging changes was detected in HPV-associated tumors, when comparing HPV-associated versus HPV non-associated groups. The mean ADC for primary tumor and metastatic lymph nodes showed a significant increase in HPV-associated group compared to baseline. Only primary tumors completely resolved during RT showed a significant rise in mean ADC compared to the baseline. In contrast, no significant changes were observed in all the studied ADC histogram parameters at different timepoints when compared to the baseline parameters in HPV non-associated cases. RPA identified a ΔADC 5th percentile > 13% at the 3rd week of RT as the most significant parameter associated with shorter time to CR for the primary tumor during RT in HPV-associated tumors. Furthermore, a significant reduction in the volume of both primary tumors and metastatic lymph nodes was observed in HPV-associated cases compared to baseline volumes at different timepoints during RT.

This study has several limitations. First, a relatively small number of patients were included in the study. Additionally, a few patients had missing images due MR-Linac technical difficulties in image acquisition earlier at the time of initial implementation of the program.

Second, the limited number of locoregional failure or recurrence events resulted in the absence of meaningful correlation between different ADC parameters, throughout the course of RT, and clinical endpoints.

Furthermore, as ours was the first prospective study to investigate the role of quantitative DWIs obtained during treatment via MR-Linac, we intended to describe the kinetics of multiple ADC parameters at regular intervals and correlate these parameters with the overall outcome of patients with HNSCC. Therefore, our cohort constituted a heterogenous group of HNSCC with different primary sites of origin, representing another limitation of our study.

Finally, small primary tumors (i.e., T1 glottic carcinoma) and small size metastatic lymph nodes were included in the study. Small ROIs might have led to bias in measurement and extraction of ADC parameters, and consequently, bias in correlation between these ADC parameters and other endpoints.

## Conclusion

Our initial experience with serial DWI acquisition during RT using the MR-Linac showed that dynamic ADC changes, for both the primary tumor and nodal disease, assessed at regular intervals during RT can be a promising biomarker for predicting early response to treatment in HNSCC patients. This may allow intra-treatment dose modification and therefore, establish the basis for quantitative image-guided dose adaptation. Further studies with larger cohorts of patients and more multi-institutional data, are needed to validate our results.

## Funding

Efforts by Drs. Fuller, Christodouleas, Dresner, Mohamed, Naser, He, Mulder, and Preston are supported by NIH National Institute of Dental and Craniofacial Research (NIDCR) Academic Industrial Partnership Grant (R01DE028290) and the Administrative Supplement to Support Collaborations to Improve AIML-Readiness of NIH-Supported Data (R01DE028290-04S2). Dr. El-Habashy received prior salary support from the Egyptian Ministry of Higher Education. Drs. McDonald and Wahid received prior salary support during study execution from the NIDCR Ruth L. Kirschstein National Research Service Award (NRSA) Individual Predoctoral Fellowship Program (F31DE029093, F31DE031502). Drs. McDonald, Wahid, and Fuller receive related salary support from the NCI Ruth L. Kirschstein National NRSA Institutional Research Training Grant Award via the MD Anderson Image Guided Cancer Therapy Training Program (T32CA261856). Dr. Jillian Rigert Receives salary support from the NIDCR Diversity Supplement (3R01DE028290-02S1). Dr. Yang receive unrelated funding from Radiation Oncology Institute. Drs. Fuller, Mohamed, and Lai receive unrelated funding from the NIDCR Establishing Outcome Measures for Clinical Studies of Oral and Craniofacial Diseases and Conditions award (R01DE025248). Dr. Fuller received/receives unrelated funding and salary support from: NSF/NIH Interagency Smart and Connected Health (SCH) Program (R01CA257814); NIH National Institute of Biomedical Imaging and Bioengineering (NIBIB) Research Education Programs for Residents and Clinical Fellows Grant (R25EB025787); NIH NIDCR Exploratory/Developmental Research Grant Program (R21DE031082); NIH/NCI Cancer Center Support Grant (CCSG) Pilot Research Program Award from the UT MD Anderson CCSG Radiation Oncology and Cancer Imaging Program (P30CA016672); Patient-Centered Outcomes Research Institute (PCS-1609-36195) sub-award from Princess Margaret Hospital; National Science Foundation (NSF) Division of Civil, Mechanical, and Manufacturing Innovation (CMMI) grant (NSF 1933369). Dr. Fuller receives grant and infrastructure support from MD Anderson Cancer Center via: the Charles and Daneen Stiefel Center for Head and Neck Cancer Oropharyngeal Cancer Research Program; the Program in Image-guided Cancer Therapy; and the NIH/NCI Cancer Center Support Grant (CCSG) Radiation Oncology and Cancer Imaging Program (P30CA016672). Dr. Fuller has received direct industry grant/in-kind support, honoraria, and travel funding from Elekta AB. Dr. Fuller has received travel, speaker honoraria and/or registration fee waiver unrelated to this project from: The American Association for Physicists in Medicine; the University of Alabama-Birmingham; The American Society for Clinical Oncology; The Royal Australian and New Zealand College of Radiologists; The American Society for Radiation Oncology; The Radiological Society of North America; and The European Society for Radiation Oncology.

## CRediT authorship contribution statement

**Dina M. El-Habashy:** Formal analysis, Methodology, Writing – original draft, Visualization, Writing – review & editing. **Kareem A. Wahid:** Software, Data curation, Writing – review & editing. **Renjie He:** Software, Writing – review & editing. **Brigid McDonald:** Writing – review & editing. **Jillian Rigert:** Writing – review & editing. **Samuel J. Mulder:** Writing – review & editing. **Tze Yee Lim:** Writing – review & editing. **Xin Wang:** Writing – review & editing. **Jinzhong Yang:** Writing – review & editing. **Yao Ding:** Methodology. **Mohamed A. Naser:** Data curation. **Sweet Ping Ng:** Writing – review & editing. **Houda Bahig:** Writing – review & editing. **Travis C. Salzillo:** Writing – review & editing. **Kathryn E. Preston:** Resources. **Moamen Abobakr:** Writing – review & editing. **Mohamed A. Shehata:** Supervision, Writing – review & editing. **Enas A. Elkhouly:** Supervision, Writing – review & editing. **Hagar A. Alagizy:** Supervision, Writing – review & editing. **Amira H. Hegazy:** Supervision, Writing – review & editing. **Mustefa Mohammadseid:** Resources. **Chris Terhaard:** Writing – review & editing. **Marielle Philippens:** Writing – review & editing. **David I. Rosenthal:** Writing – review & editing. **Jihong Wang:** Writing – review & editing. **Stephen Y. Lai:** Writing – review & editing. **Alex Dresner:** Writing – review & editing. **John C. Christodouleas:** Funding acquisition, Writing – review & editing. **Abdallah Sherif Radwan Mohamed:** Funding acquisition, Conceptualization, Supervision, Writing – review & editing. **Clifton D. Fuller:** Funding acquisition, Conceptualization, Supervision, Writing – review & editing.

## Declaration of Competing Interest

The authors declare the following financial interests/personal relationships which may be considered as potential competing interests: C.D.F. has received direct industry grant support, speaking honoraria, and travel funding from Elekta AB. The other authors have no conflicts of interest to disclose.

## Footnotes

Appendix A Supplementary data to this article can be found online at https://doi.org/10.1016/j.ctro.2023.100666.
