# Supplementary material for: Longitudinal diffusion and volumetric kinetics of head and neck cancer magnetic resonance on a 1.5 T MR-linear accelerator hybrid system: A prospective R-IDEAL stage 2a imaging biomarker characterization/pre-qualification study
Source: Clin Transl Radiat Oncol. 2023 Jul 24;42:100666. doi: 10.1016/j.ctro.2023.100666 (PMC10424120; doi:10.1016/j.ctro.2023.100666)
Supplement: Supplementary data 1 [file mmc1.docx]

**Appendix A**

**Details of MRI sequences acquisition parameters.**

The following images were collected:

1. Three-dimensional (3D) T2-weighted MRI, which was used for image registration during daily treatment (repetition time = 1535 ms, echo time = 278 ms, pixel bandwidth = 740 Hz, flip angle = 90°, echo train length = 114, field of view = 400×400×300 mm^3^, reconstructed voxel size = 0.83×0.83×1 mm^3^, scan time = 2 minutes, number of average = 1, and SENSE factor = 4) (172 scans)

2. 3D T2-weighted MRI without fat suppression, which was used for target segmentation (repetition time = 2100 ms, echo time = 375 ms, pixel bandwidth = 459 Hz, flip angle = 90°, echo train length = 150, field of view = 520×520×300 mm^3^, and reconstructed voxel size = 0.98×0.98×2.2 mm^3^, scan time = 6 minutes, number of average = 2, and SENSE factor = 2) (9 scans)

2. 3D T2-weighted MRI with fat suppression, which was used for target segmentation (repetition time = 1400 ms, echo time = 190 ms, pixel bandwidth = 473 Hz, flip angle = 90°, echo train length = 76, field of view = 520×520×300 mm^3^, and reconstructed voxel size = 0.98×0.98×1.2 mm^3^, scan time = 6 minutes, fat saturation = SPAIR, number of average = 2, and SENSE factor = 2) (1 scan)

3. Single-shot echo planar DWI covering targets and organs-at-risk, which was used for treatment assessment (b values = 0, 150, and 500 s/mm^2^; repetition time = 5700 ms; echo time = 75 ms; pixel bandwidth = 2174 Hz;  flip angle = 90°; echo train length = 39; field of view = 300×300×158 mm^2^; reconstructed voxel size = 1.6×1.6×1.3 mm^3^; scan time = 3 minutes; fat saturation = SPAIR; and SENSE factor = 2.2). In our study, we adhered to the consensus EPI protocol that had been distributed among the MR-linac Consortium [18].

4. ADC maps were reconstructed using only the b-values of 150 and 500 s/mm^2^; the b=0 images were excluded to minimize the effects of perfusion on ADC calculations and to be consistent with MR-Linac Consortium recommendations [8]. These ADC maps were subsequently used to extract the histogram parameters of the segmented regions of interest (ROIs).
