# Supplementary material for: Longitudinal diffusion and volumetric kinetics of head and neck cancer magnetic resonance on a 1.5 T MR-linear accelerator hybrid system: A prospective R-IDEAL stage 2a imaging biomarker characterization/pre-qualification study
Source: Clin Transl Radiat Oncol. 2023 Jul 24;42:100666. doi: 10.1016/j.ctro.2023.100666 (PMC10424120; doi:10.1016/j.ctro.2023.100666)
Supplement: Supplementary data 2 [file mmc2.docx]

**Appendix B**

**HPV-associated Vs HPV non-associated tumors**

We analyzed the kinetics of ADC changes for the HPV-associated versus HPV non-associated patients to demonstrate the specific profile of ADC changes in each cohort.

There were 22 patients with HPV-associated and 8 patients with HPV non-associated patients included in this study. In HPV-associated group, half of the primary tumors completely resolved during RT, while none of the primary tumors in the HPV non-associated group achieved CR during treatment (p = 0.014). Patients’ and disease characteristics for HPV-associated and HPV non-associated are summarized in tables B.1 and B.2.

In HPV-associated group, there was a significant increase in mean ADC (p = 0.018, 0.002, 0.001, 0.004 and 0.007 for both GTV-P & p = < 0.001, 0.001 and 0.009 for GTV-N) compared to baseline mean ADC. This incremental increase in the GTV-P mean ADC was statistically significant (p = 0.04, 0.006, 0.004, 0.04 and 0.016, respectively) only for primary tumors developing CR intra-treatment (n = 11, 50%). In contrast, no significant changes in the GTV-P mean ADC was detected compared to the baseline values, in the HPV non-associated group (p > 0.05).

For the HPV-associated group, RPA identified a ΔADC 5^th^ percentile > 13% at the 3rd week of RT as the most significant parameter associated with shorter time to CR for the primary tumor during RT (p = 0.001).

There was a significant decrease in residual tumor volumes for both GTV-P (p = 0.006 and < 0.001, respectively) and GTV-N (p = 0.04, 0.001, 0.001, and < 0.001, respectively) compared to baseline volumes at different timepoints throughout the RT course, in the HPV+ group.

For both groups, no statistically significant correlation was found between GTV-P or GTV-N ΔADC parameters at various timepoints and development of either CR (n = 18, 81.8% for HPV-associated and n = 8, 100% for HPV non-associated) or recurrence (n = 3, 13.6% for HPV-associated and n = 2, 25% for HPV non-associated) after the end of RT (p > 0.05 for all).

**Table B.1. HPV-associated Patients’ and Disease Characteristics:**

| **Patient characteristics** | **Mean ± SD, range** |
| --- | --- |
| **Age (years)** | 65.43 ± 9.98, 37-81 |
| **Gender**  Male  Female | 21 (95.45%)  1 (4.54%) |
| **Primary sites**  Tonsil  BOT  CUP  Larynx | 11 (50%)  9 (40.9%)  1 (4.54%)  1 (4.54%) |
| **TNM Stage**  I  II  III  IV | 15 (68.2%)  2 (9%)  3 (13.6%)  2 (9%) |
| **T stage**  Tx  T1  T2  T3  T4 | 1 (4.54%)  7 (31.8%)  11 (50%)  1 (4.54%)  2 (9%) |
| **N stage**  N0  N1  N2  N3 | 2 (9%)  14 (63.63%)  5 (22.7%)  1 (4.54%) |
| **Smoking Status**  Non-smoker  Ex-smoker | 12 (54.54%)  10 (47.6%) |
| **Surgery for the primary**  Yes  No | 7 (31.8%)  15 (68.2%) |
| **RT dose (Gy)** | 69.55 ± 1.19, 66-70 |
| **Number of fractions** | 32.8 ± 1.03, 30-35 |
| **Treatment**  Radiation therapy alone  Concurrent Chemotherapy | 6 (27.3%)  16 (72.7%) |
| **CR for the primary during RT course**  Yes  No | 11 (50%)  11 (50%) |
| **CR at the end of RT**  Yes  No | 18 (81.8%)  4 (18.2%) |
| **Recurrence**  Yes  No | 3 (13.6%)  19 (86.36%) |
| **Type of recurrence**  Local and/ or regional  Distant | 0  3 |

**Table B.2. HPV non-associated Patients’ and Disease Characteristics:**

| **Patient characteristics** | **Mean ± SD, range** |
| --- | --- |
| **Age (years)** | 61/38 ± 11.83, 42-82 |
| **Gender**  Male  Female | 7 (87.5%)  1 (12.5%) |
| **Origin**  Larynx  Oropharynx  Hypopharynx | 6 (75%)  1 (12.5%)  1 (12.5%) |
| **TNM Stage**  I  II | 6 (75%)  2 (25%) |
| **T stage**  T1  T2 | 6 (75%)  2 (25%) |
| **N stage**  N0  N2 | 7 (87.5%)  1 (12.5%) |
| **Smoking Status**  Smoker  Non-smoker  Ex-smoker | 1 (12.5%)  3 (37.5%)  4 (50%) |
| **Surgery for the primary**  Yes  No | 0  8 (100%) |
| **RT dose (Gy)** | 65.67 ± 2.89, 63-69.96 |
| **Number of fractions** | 29 ± 2.41, 28-33 |
| **Treatment**  Radiation therapy alone  Induction Chemotherapy  Concurrent Chemotherapy | 5 (62.5%)  1 (12.5%)  2 (25%) |
| **CR for the primary during RT course**  Yes  No | 0  8 (100%) |
| **CR at the end of RT**  Yes  No | 8 (100%)  0 |
| **Recurrence**  Yes  No | 2 (25%)  6 (75%) |
| **Type of recurrence**  Local  Regional  Distant | 1 (12.5%)  0  1 (12.5%) |
| **Gross tumor disease**  GTV-P only  GTV-P & GTV-N  GTV-N only | 7 (87.5%)  8 (100%)  1 (12.5%) |


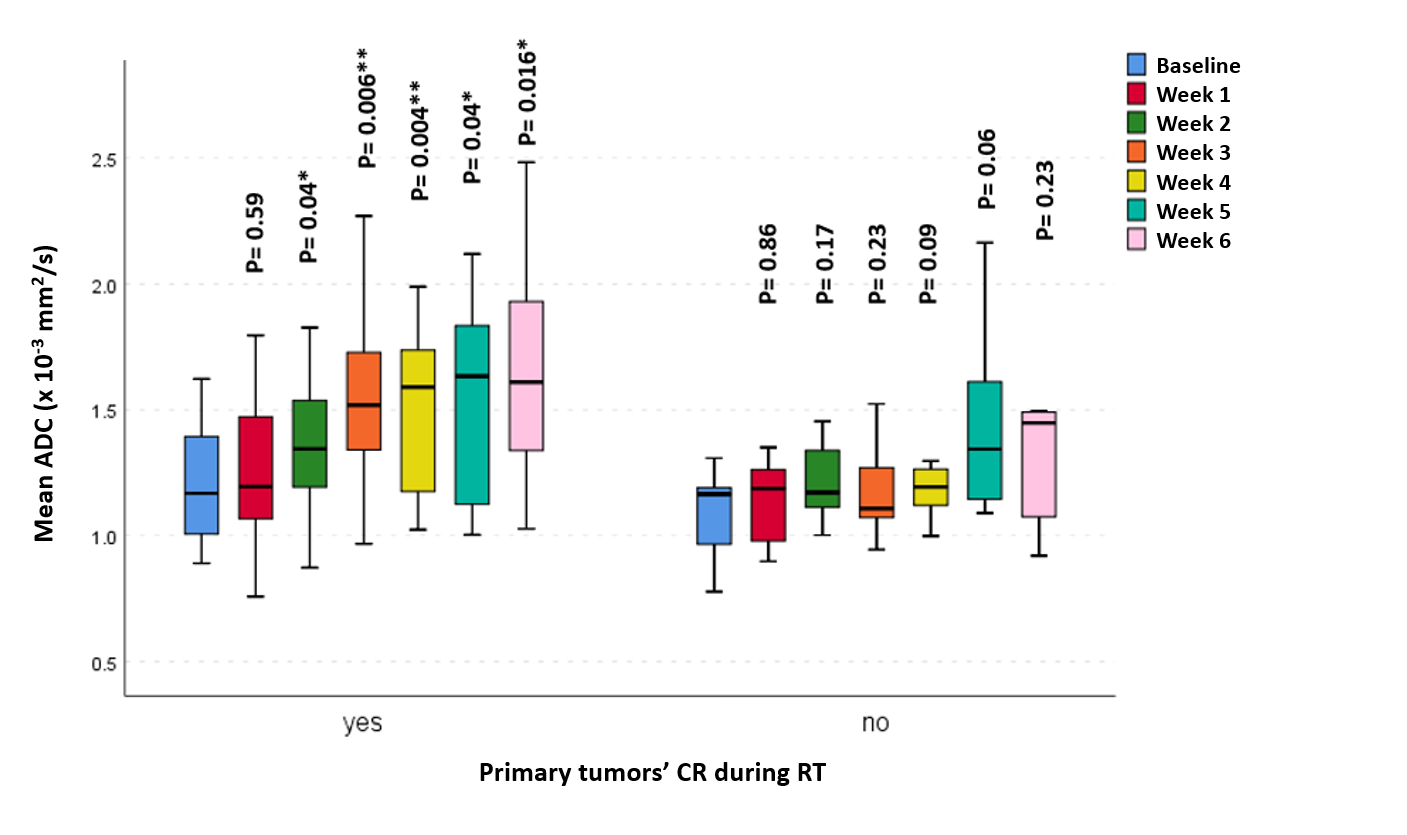


Figure B.1. Mean apparent diffusion coefficient (ADC) at different timepoints for primary tumors which developed CR during RT versus those did not (HPV-associated).

*Significance before Bonferroni correction

** Significance after Bonferroni correction


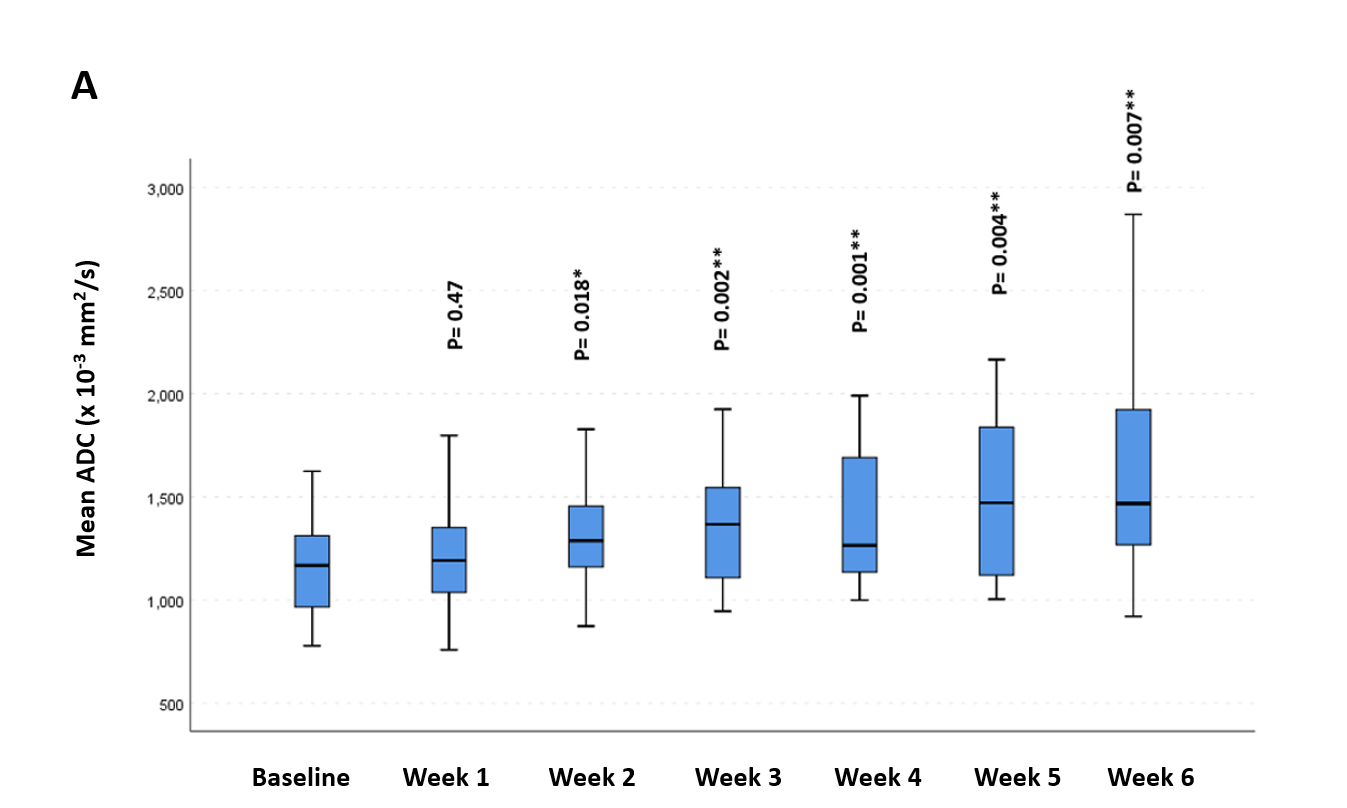


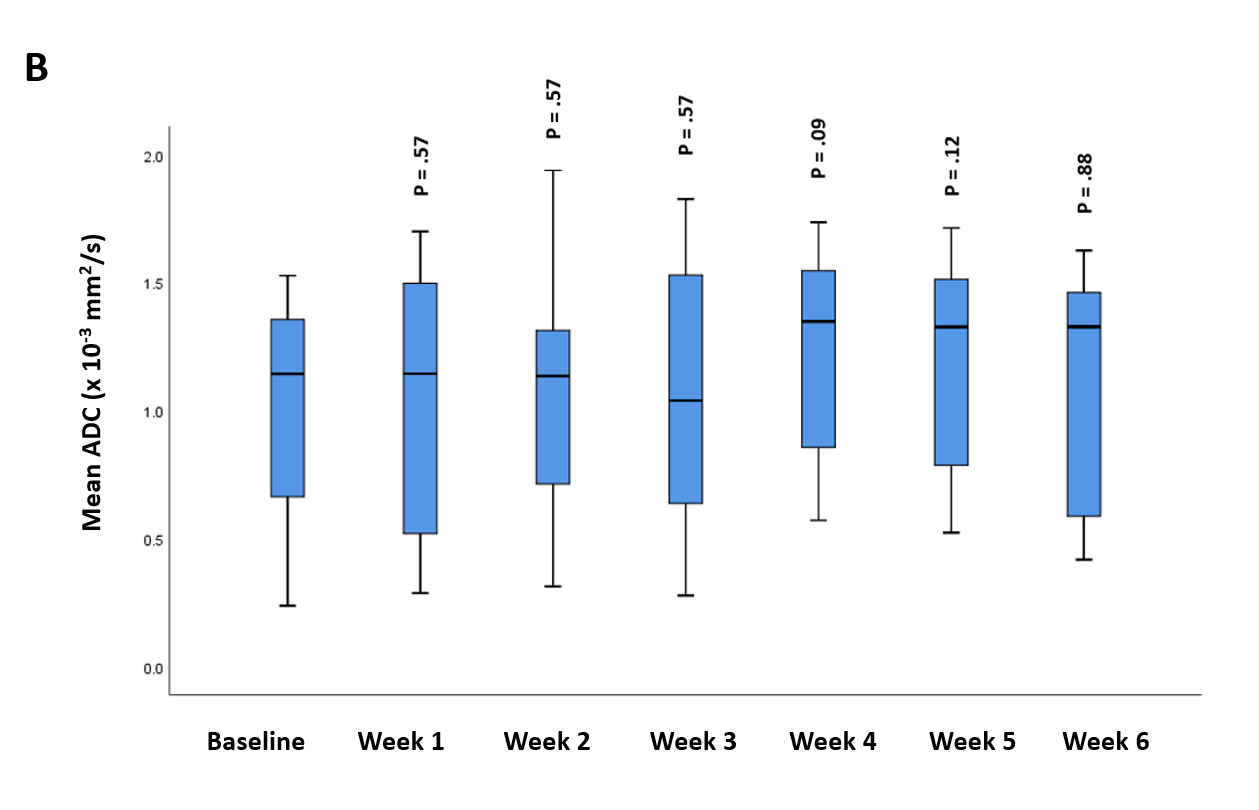


Figure B.2. Mean ADC at different timepoints for GTV-P: (A) HPV-associated, (B) HPV non-associated

*Significance before Bonferroni correction

** Significance after Bonferroni correction


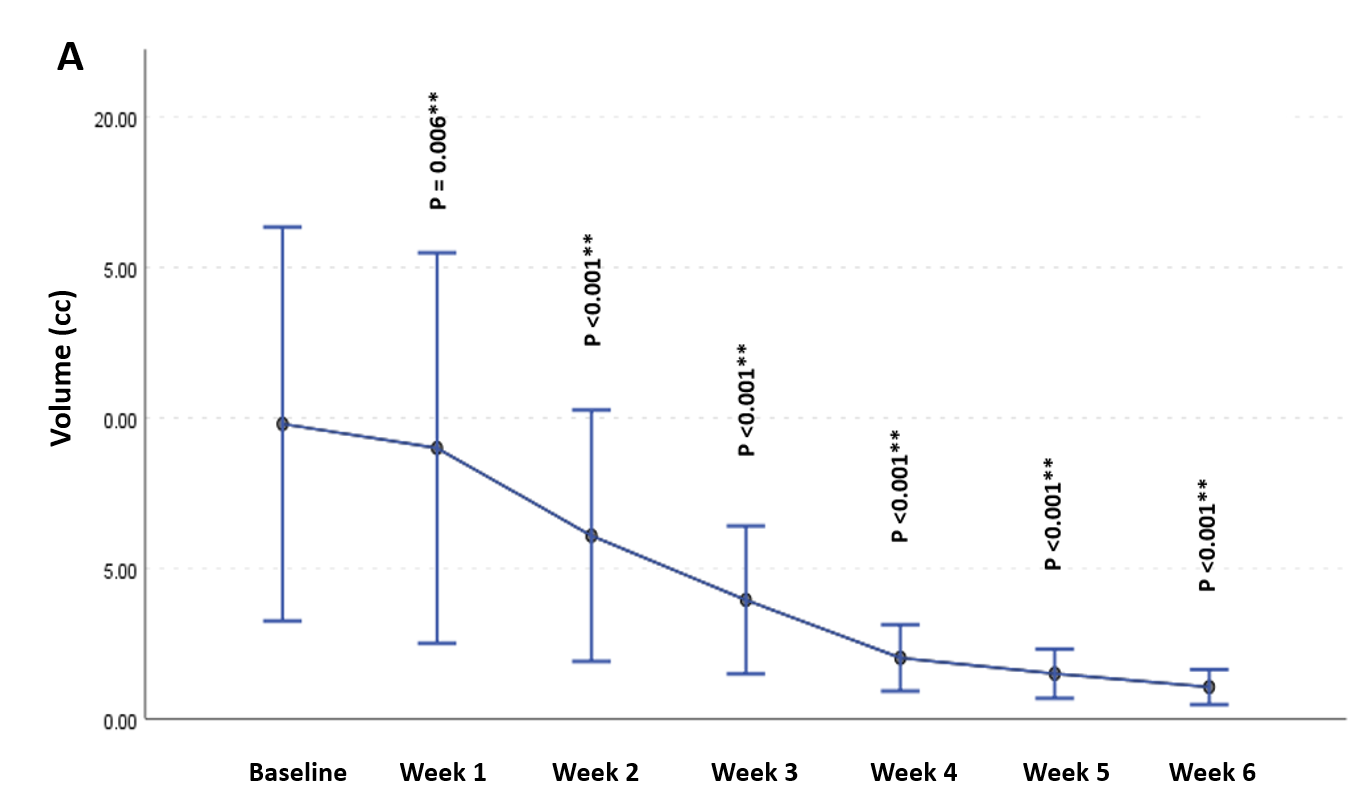


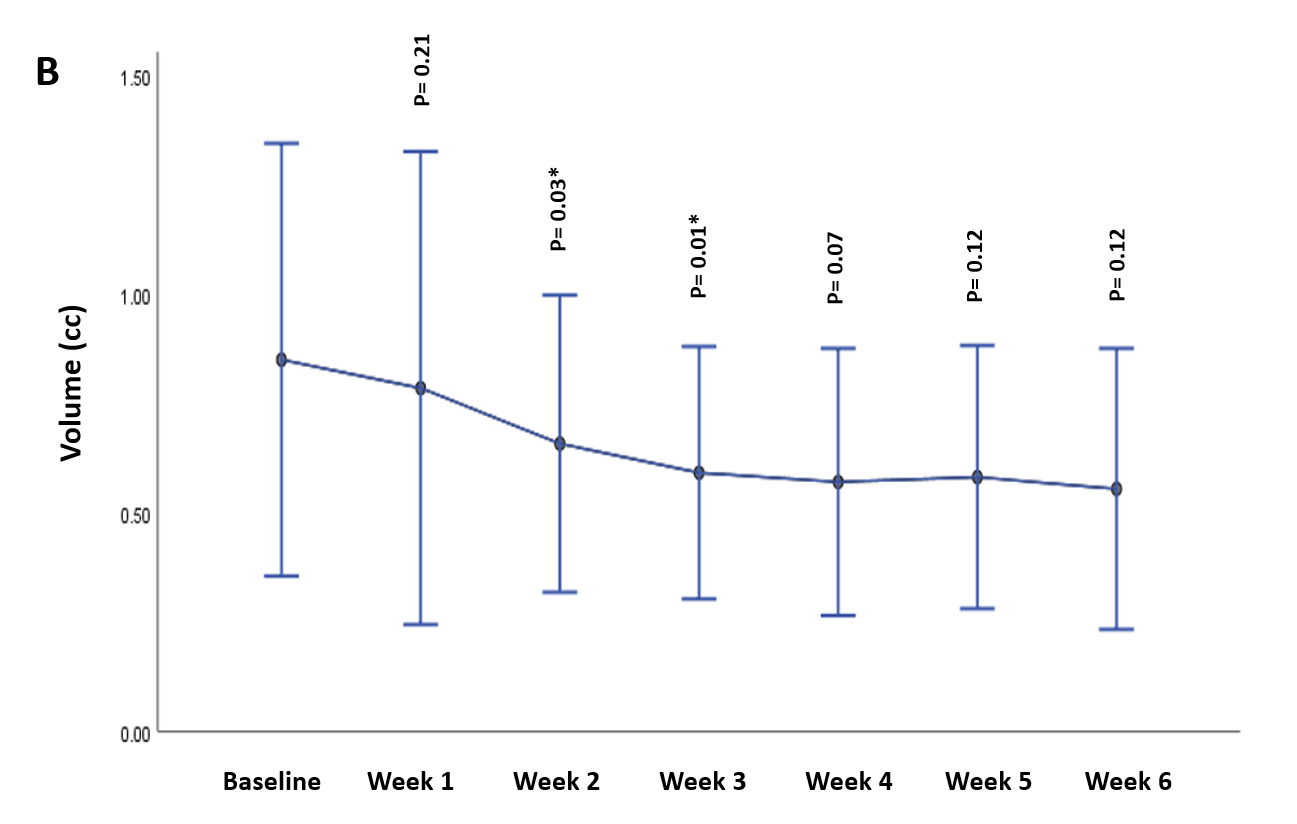


Figure B.3. Volumetric changes in GTV-P throughout the course of radiation therapy: (A) HPV-associated cases, (B) HPV non-associated

*Significance before Bonferroni correction

** Significance after Bonferroni correction


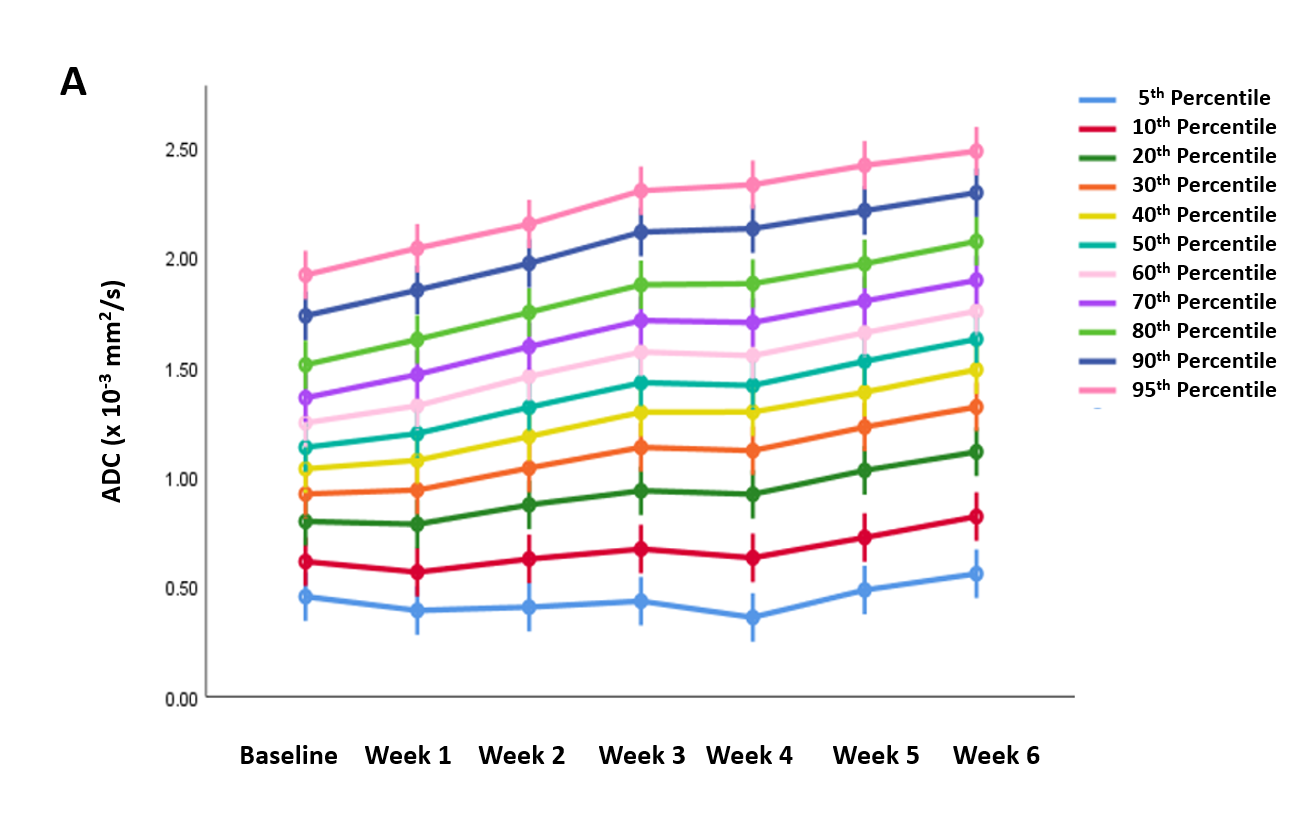


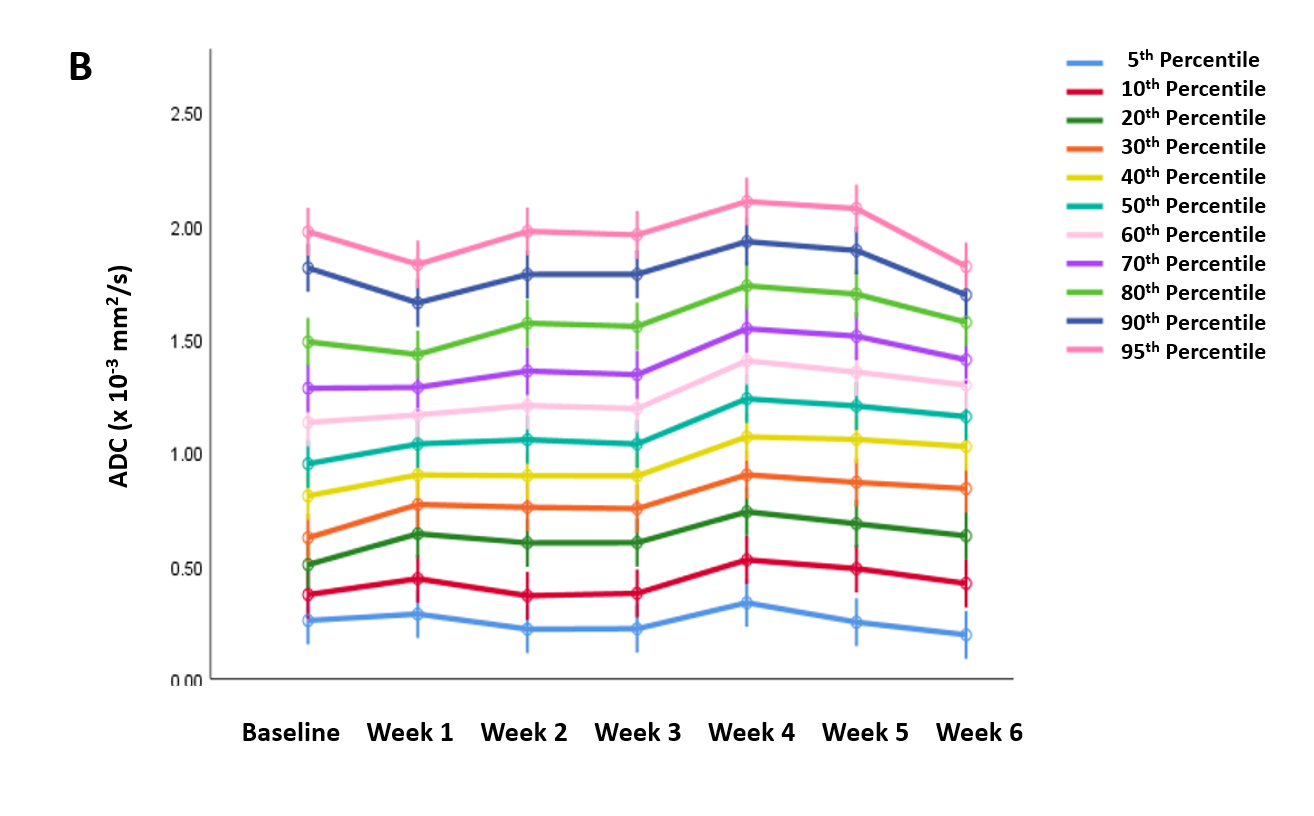


Figure B.4. Absolute ADC histogram parameters for GTV-P across different time points: (A) HPV-associated, (B) HPV non-associated.


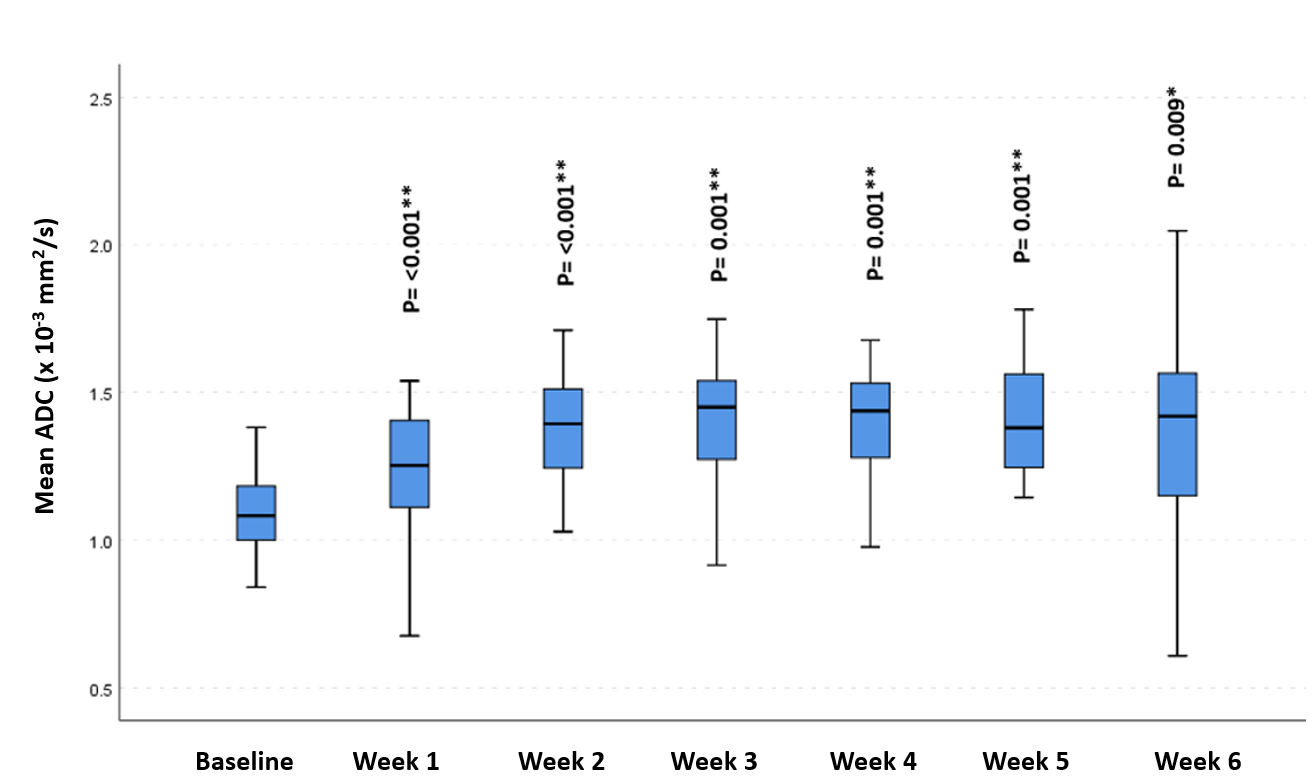


Figure B.5. Mean ADC at different timepoints for GTV-N in HPV-associated cases

*Significance before Bonferroni correction

** Significance after Bonferroni correction


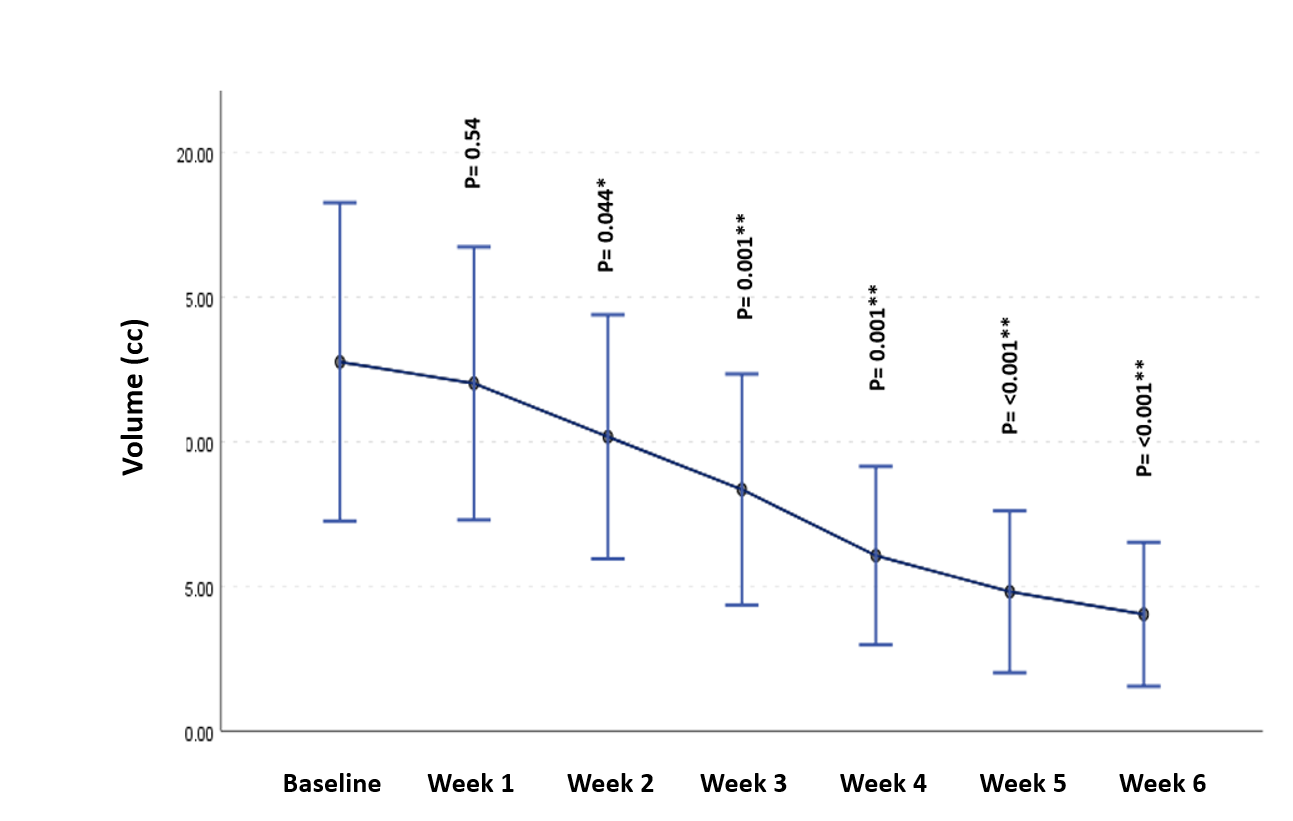


Figure B.6. Volumetric changes in GTV-N throughout the course of radiation therapy in HPV-associated cases

*Significance before Bonferroni correction

** Significance after Bonferroni correction
